# Addressing “Nature-Deficit Disorder”: A Mixed Methods Pilot Study of Young Adults Attending a Wilderness Camp

**DOI:** 10.1155/2015/651827

**Published:** 2015-12-16

**Authors:** Sara L. Warber, Ashley A. DeHudy, Matthew F. Bialko, Melissa R. Marselle, Katherine N. Irvine

**Affiliations:** ^1^Department of Family Medicine, University of Michigan Medical School, 1018 Fuller Street, Ann Arbor, MI 48104, USA; ^2^European Centre for Environment and Human Health, University of Exeter, Truro TR1 3HD, UK; ^3^Department of Pediatrics, University of Michigan Medical School, 1500 E. Medical Center Drive, Ann Arbor, MI 48109, USA; ^4^School of Medicine, St. George's University, University Centre, West Indies, Grenada; ^5^Institute of Energy and Sustainable Development, De Montfort University, Leicester LE1 9BH, UK; ^6^School of Arts & Media, University of Salford, Salford M6 3EQ, UK; ^7^Social, Economic and Geographical Sciences Research Group, James Hutton Institute, Craigiebuckler, Aberdeen AB15 8QH, UK

## Abstract

*Background and Objectives*. Rapid urbanization raises concern about chronic human health issues along with less frequent interaction with the natural world. “Nature-deficit disorder,” a nonclinical term, describes this potential impact on the well-being of youth. We conducted a mixed methods pilot study of young adults attending a four-week wilderness camp to investigate whether nature-based camp experiences would increase connection to nature and promote multiple dimensions of well-being.* Methods*. Participants completed precamp (*n* = 46) and postcamp (*n* = 36) online questionnaires including nature-related and holistic well-being measures. Differences were investigated using paired* t*-tests. Interviews (*n* = 16) explored camp experiences and social relations.* Results*. All nature-related measures—exposure, knowledge, skills, willingness to lead, perceived safety, sense of place, and nature connection—significantly increased. Well-being outcomes also significantly improved, including perceived stress, relaxation, positive and negative emotions, sense of wholeness, and transcendence. Physical activity and psychological measures showed no change. Interviews described how the wilderness environment facilitated social connections.* Conclusion*. Findings illustrate the change in nature relations and well-being that wilderness camp experiences can provide. Results can guide future research agendas and suggest that nature immersion experiences could address the risk of “nature-deficit disorder,” improve health, and prepare future environmental leaders.

## 1. Introduction

With over half the world's population living in urbanized areas [1] there are increasing concerns over the quality of life for urban residents. This concern focuses on both noncommunicable diseases (e.g., cardiovascular) [2], mental health [3], and the loss of opportunity to interact with the natural environment [4, 5]. Louv [6], journalist and author of* Last Child in the Woods*, proposes that young people today are at risk for a nonclinical entity that he terms “nature-deficit disorder” (NDD). Louv argues that elements of our urbanized lifestyle, including fewer natural spaces, a car-focused culture, more screen time, changes in the perception of risk (e.g., “stranger danger”), less leisure time, and increased time pressures from work or school, combine to decrease or even eliminate contact with nature for both adults and children. He proposes that direct exposure to nature is essential for the physical and emotional health of both children and adults. “Nature-deficit disorder” is not yet regarded as a medical condition—it is not recognized by any medical coding schemes, such as ICD-10 [7] or the DSM-5 [8, 9], the American Psychiatric Association's classification and diagnostic tool. However, Louv's work draws on theory that exposure to the natural environment can be cognitively restorative [10, 11], reduces stress [12], and promotes a sense of place [13, 14]. Empirical evidence from numerous fields of study, including environmental psychology [15] landscape architecture [16], and public health [17], supports these ideas and also suggests that human well-being and global chronic health issues (e.g., stress, depression, and cardiovascular disease) may improve through interaction with nature [18, 19].

Previous research focused on youth and young adults demonstrates that exposure to nature improves cognitive functioning [20], decreases attention deficit disorder [21], and promotes self-awareness [22]. Meta-analysis demonstrates that wilderness challenge programs can have medium effect sizes on delinquent behavior and other measures of psychological well-being [23]. Research into camping experiences demonstrates effects on aspects of character development, such as self-confidence, self-esteem, and social relationships [24]. Questions remain however regarding the relationship of youth nature camp experiences to measures of health and well-being.

Recent research into nature contact in urban green places suggests that participants' benefits map onto the holistic biopsychosocial-spiritual model of health [25, 26]. The biopsychosocial model [27, 28] expands the biomedical model by including psychological health and social support. With increasing recognition of a spiritual dimension, some argue for a biopsychosocial-spiritual model [29]. Additionally, research on the construct of psychological well-being identifies both a cognitive and an affective portion [30]. Qualitative research indicates that benefits from nature contact may include physical relaxation, mental restoration, positive emotions toward self and place, social connectedness, and experiences of tranquility and peace [31]. Numerous studies have explored nature's impact on these dimensions of health including general health [32, 33], physiologic effects [18, 34], perceived stress and emotional well-being [15], social relations [35], and transcendent experiences [36, 37]. Although these examples in sum indicate effects of nature on several dimensions of health and well-being, few have purposefully evaluated the health effects of nature using a broad biopsychosocial-spiritual framework.

There is growing interest in camps and camping as a way of increasing nature exposure and addressing “nature-deficit disorder” [38]. However, few studies have directly examined this idea. We, therefore, sought to conduct a pilot study evaluating the effects of a four-week immersion in nature in a residential camp setting for young adults. We hypothesized that the experience would promote greater comfort and connection with nature as well as improved physical, psychological, emotional, social, and spiritual well-being among both campers and staff.

## 2. Methods

We utilized a mixed-methods design that incorporated a pre-post within-subjects assessment employing online questionnaires as well as face-to-face interviews. Ethical approval was received by the University of Michigan's Medical Institutional Review Board.

### 2.1. Study Setting

The National Youth Science Camp (NYSC) is a 4-week residential science education camp held in the rural mountains of West Virginia, USA, in a cell phone-free area (i.e., the 13,000 sq mile (33,000 sq km) US Radio Quiet Zone). Although cell phones are not available, very limited internet connection (i.e., slow, sometimes unavailable) is available for computers used in educational opportunities (e.g., computer modeling, building computers, and writing code/programming) and for communicating with those at home (email, social media). The camping program incorporates lectures, hands-on studies, and opportunities to participate in outdoor adventure activities.

### 2.2. Recruitment and Consent

Each state competitively selects two delegates to attend the camp in the summer following graduation from high school; delegates occasionally attend from other countries. Selection is based on academic achievement, leadership, and demonstrated interest in the sciences; attendance is free of charge. Camp staff members are typically former campers.

All delegates (“campers”) and staff members (“staff”) (18 years old or older) received a postal letter explaining the study followed by an email invitation with a web link to an online precamp questionnaire which incorporated a consent statement (i.e., comprehensive oral consent). Weekly email reminders were sent during the month prior to camp until the potential participant joined the study, declined participation, or opted out of receiving further emails.

A subset of study volunteers who had indicated willingness to be interviewed (via the precamp questionnaire) were invited to participate in individual, in-depth interviews conducted* in situ* during the camp. The subset sample comprised participants purposively chosen to reflect the diversity of the larger sample along the following dimensions: camper or staff, gender, baseline nature connection score, and baseline nature experience score. A comprehensive written consent was signed prior to interview.

### 2.3. Assessment

All questionnaires were web-based. Precamp questionnaires were completed prior to arrival with postcamp surveys completed during the last week of camp or the week of returning home; the response window was lengthened due to the limited Internet capability at the camp. Two multifaceted topic areas of relevance were explored: nature-related and well-being. Where available, we sought to utilize theory and/or validated measures; here we provide brief descriptions of the individual measures.

### 2.4. Nature-Related Measures

Four topic areas were specifically included to examine the experience of and relationship with the natural environment component of the camp experience (Table 1). Nature experience measures were modified from previous research and focused on exposure (6 items, e.g., during nonschool/nonwork time, how often do you observe nature; 1 = never, 5 = very frequently [39]); knowledge (5 items, e.g., “how much knowledge would you say you have about native plants?”; 1 = none at all, 5 = a great deal; modified from [40]); skill (17 items, e.g., “how skilled in fire building would you say that you are?”; 1 = not at all skilled, 6 = extremely skilled); and leadership (i.e., “would you be likely to volunteer to take charge of a camping trip in a wilderness area?”; 1 = not at all, 6 = extremely likely). Measures for the latter two areas were developed based on questions from Kaplan's [41] study of an outdoor challenge program and on insight into specific NYSC wilderness-related activities. For each measure, a mean response was calculated for use in analysis; higher values suggest more exposure to, knowledge of, skills related to and likelihood of taking a leadership role for activities in nature.

The three remaining nature-related topics focused on safety, sense of place, and connection to nature. The degree to which one felt safe in the natural environment was assessed with a single statement (“I feel safe in the natural environment”; 1 = strongly disagree, 5 = strongly agree), a measure used in a previous study on the experience of urban nature [42]. Sense of place, specifically an individual's attachment to and sense of continuity of self across time from the natural environment, was measured using scales from previous studies of urban nature [43]. Participants indicated agreement (1 = strongly disagree; 5 = strongly agree) with statements (e.g., “the natural environment feels almost like a part of me”; “I gain pleasure from being in the natural environment”) about being in the natural environment. Nature connection was measured using a 14-item validated scale [44] with participants indicating the extent to which they agree (1 = strongly disagree; 5 = strongly agree) with several statements (e.g., “I often feel part of the web of life”; “my personal welfare is independent of the welfare of the natural world”). Nature connection has previously been used with young adults [44]. Final scores for the two sense of place scales and the connection to nature scale were obtained by computing a mean following reverse coding of items as needed.

### 2.5. Well-Being Measures

Multiple aspects of well-being, corresponding to the biopsychosocial-spiritual model of health [27–29], were assessed (Table 2). Physical activity was assessed using a single question: “In the last seven days on how many days have you done a total of 30 minutes or more of physical activity, which was enough to raise your breathing rate?” [45] Responses were recorded on an 8-point scale (0 = 0 days; 7 = 7 days) and the question has been previously used in nature-health research [46]. A single-item statement was developed which asked participants “Have you felt relaxed?” during the last week (1 = all of the time; 5 = none of the time); the stem question was drawn from the SF-12 [46]. This item was reverse-scored for analysis.

Psychological domains of well-being included perceived stress, psychological well-being, self-esteem, resilience, self-awareness, and the ability to reflect. The 10-Item Perceived Stress Scale [47] measured perceptions of stress. Participants rated the frequency of experiencing certain thoughts and feelings in the past two weeks (0 = never; 4 = very often). Total scores range from 0 to 40; higher scores indicate greater psychological stress. This measure has been used in previous nature and health studies [15, 48, 49].

A global sense of psychological well-being was measured using fifteen items (this consisted of 3-item scales for five of the six subconstructs. While we appreciate that the 3-item scales have low internal consistency, we selected to use these in order to reduce participant burden) from the Ryff Psychological Well-Being Scale [50]. Participants indicated agreement (1 = strongly disagree; 6 = strongly agree) and total sum scores were created, ranging from 15 to 90 with higher scores suggesting greater psychological well-being. Self-esteem was measured using the 10-Item Rosenberg Self-Esteem Scale [51]. Responses (0 = strongly disagree; 3 = strongly agree) were provided to a set of statements “dealing with your general feelings about yourself” (e.g., “I take a positive attitude toward myself”; “I am inclined to feel that I am a failure”) and summed across statements producing a range of 0 to 30; scores below 15 suggest low self-esteem, while those between 15 and 25 are within the normal range.

The Ego-Resiliency Scale [52] assessed resilience. Participants indicate agreement with 14 statements (e.g., “I quickly get over and recover from being startled”; 1 = disagree very strongly, 4 = agree very strongly). From the Situational Self-Awareness Scale [53] we used the 3-Item Public Self-Awareness Subscale (e.g., “I am concerned about the way I present myself”; 1 = strongly disagree, 7 = strongly agree); this scale assesses “how you feel right now, at this instant.” The last aspect of psychological well-being focused on reflection, an idea drawn from the literature on the cognitively restorative benefits of interaction with the natural environment (e.g., [10]). A modified 3-item (e.g., you have perspective on life) scale was developed from previous nature-health studies [39, 40, 42]. Participants were asked: “at this point in your life, to what extent do you feel…” with responses provided on a 1 (not at all) to 5 (almost always) response scale. Final scores for these three scales were obtained by computing a mean following reverse coding of items as needed. For each, a high score indicates greater resilience, self-awareness, and reflection.

To assess emotional state, the Positive and Negative Affect Schedule (PANAS) [54] measured both positive and negative affect. Participants rated the frequency of experiencing 10 positive and 10 negative emotions in the past two weeks (1 = very slightly or not at all; 5 = extremely). For each scale higher scores demonstrate greater positive or negative affect. The PANAS has been used in previous nature and health studies [55–57].

Differences in social well-being were measured using the 14-Item Positive Relations with Others Scale [58] with responses made on a 5-point scale (1 = strongly disagree; 5 = strongly agree). After reverse scoring of selected items, possible scores range from 14 to 84 with high scores suggesting more positive relations with others.

We included two aspects related to the domain of spiritual well-being. An individual's sense of wholeness was measured using a 7-item (e.g., disconnected from what is important in life) scale from a previous study on nature and health [39]; participants indicated to what extent they felt a certain way at this point in their life (1 = strongly disagree; 5 = strongly agree). To measure transcendence we used seven items from the Mysticism Scale [59] selected based on face validity. Participants were asked to indicate how true a particular statement (e.g., “I have had an experience which I knew to be sacred”) was of their experience (1 = definitely not true; 5 = definitely true). Final scores for sense of wholeness were obtained by computing a mean (following reverse coding as needed); total scores for transcendence ranged from 7 to 35. Higher scores suggest greater sense of wholeness and transcendence.

The precamp questionnaire also included gender, age, date of birth, where from (country of origin and where on rural/urban continuum), whether being camper or staff (if staff, education, and work). In the postcamp questionnaire, participants were provided with a list of 10 elements of the camp experience (e.g., dining hall food; spending time with friends; and overnight hiking/camping trips) and asked to separately rank their top 5 (1 = top selection) in terms of enjoyment, influence, intellectual stimulation, and hardness (i.e., difficulty). There was also one open-ended question on “what surprised you most about the camp experience?”

### 2.6. Interview Data Collection

Interviews were conducted during free time at the camp in a suitably private space (e.g., medical unit or tents on the archery field where there was minimal foot traffic). An interview guide identified topics for discussion with optional probing questions. Interviews focused on relationships between the camp experience, nature, perceived stress, and well-being. Interviews were audiotaped using a digital recording device and transcribed verbatim.

### 2.7. Quantitative Analysis

Postcamp questionnaires were matched with precamp response data. Participants who did not return a postcamp questionnaire were excluded from analysis. All scale scores were calculated as sums or means depending on usual practice for the scale or similar scales. Descriptive statistics for the group were reviewed. Nonresponders were compared to responders across demographics and nature-related variables using chi-squared tests or independent *t*-tests as appropriate for the data. Pre-post differences on each measure were evaluated using paired samples *t*-tests employing pairwise deletion of cases with missing data at the scale level. Ranking questions were examined with Friedman's Rank Test. Significant results indicated a general uniformity in the order of ranking. All analyses were performed using SPSS 21.0 version software; significance level was set at *p* < 0.05. As an exploration, Pearson's correlation between the difference scores of all measures (nature-related and well-being) was calculated.

### 2.8. Qualitative Analysis

Transcripts were initially reviewed by one author (MRM) and then fully analyzed by another author (SLW) and a student team. Broad themes across all interviews were identified and emergent contradictory themes were examined. Here we report on the elements of social connection and the perceived role played by nature.

## 3. Results

### 3.1. Participants

**Figure 1:**
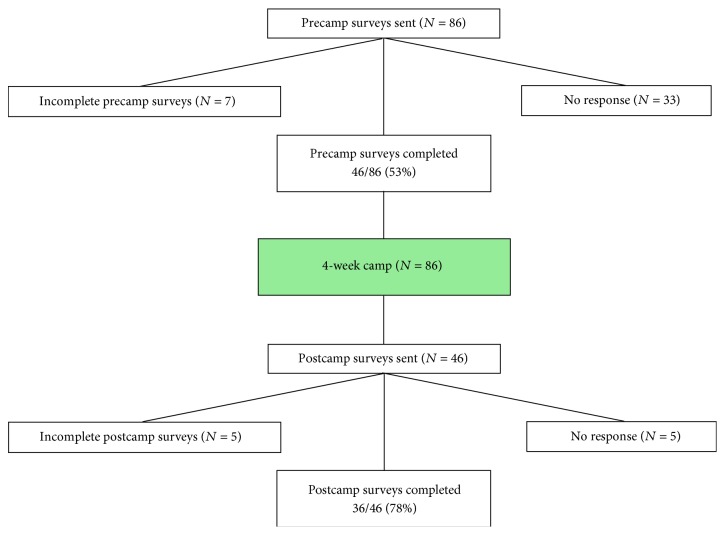
Flow diagram for study participants.

The study sample consisted of 36 campers and staff from the NYSC four-week education program in rural West Virginia who completed both precamp and postcamp questionnaires (Figure 1). Participants ranged in age from 18 to 31 years old (mode 18 years), and 67% were female (Table 3). All participants were from the United States with the exception of one from the United Kingdom. Half of the staff were current college students; the remaining had a Bachelor's degree. Nearly half (47%) of the participants identified their home environment as suburban or urban. There were no significant differences between postcamp responders and nonresponders on sociodemographics or nature-related measures.

Of the 16 participants who were interviewed, 63% were female and 56% were campers. Interviewees' age range and mean were identical to the study sample. Identification of a suburban or urban area as one's home environment was slightly higher at 56%.

### 3.2. Ranking of Camp Activities

Ranking results (*p* < 0.001 for all) show that the most enjoyable and most influential activities were similarly aligned (Table 4). Nature-based overnights and hikes ranked second only to spending time with friends as being most enjoyable and influential. Nature activities, including outdoor exercise, clustered together but ranked below several social activities as being most intellectually stimulating. The hardest activities were those that represented a change from the usual daily routine, that is, decreased computer time, rustic accommodations, the dining hall food, and overnight camping/hiking trips. The use of computers for educational activities such as modelling and the slow connection may also have contributed to it being ranked hardest of all the activities.

Further comments indicated that participants found the ranking process difficult, specifically for the most enjoyable and most intellectually stimulating categories as multiple elements of the camp experience were noted as worthy of their top selection. Most experiences were viewed positively and participants felt the components were well integrated. One participant noted, “That list does not feel entirely accurate, given there were actually very few degrees of separation in my enjoyment of all the aspects I selected. In fact, the most enjoyable part of my camp experience may have been how all the components seemed to complement each other in a way that I still do not understand, even from a more informed vantage.” Another commented, “Again, it was hard to rank, given the interconnectedness of the camp's aspects.”

### 3.3. Nature-Related Measures

All nature measures (Table 5) showed a significant change from precamp to postcamp for this group of campers and staff. Their experience with nature was markedly increased as measured by changes in exposure (*t*(35) = −5.25, *p* < 0.001), skills (*t*(35) = −6.61, *p* < 0.001), knowledge (*t*(35) = −2.47, *p* = 0.018), and willingness to lead in a natural setting (*t*(33) = −5.42, *p* < 0.001). Importantly, their sense of feeling safe in a natural environment increased as well (*t*(35) = −5.92, *p* < 0.001). Participants' sense of place, consisting of both emotional attachment to place (*t*(35) = −2.20, *p* = 0.035) and a sense of continuity with the past (*t*(35) = −3.16, *p* = 0.003), also increased. Finally, both campers and staff showed a greater sense of connection to nature (*t*(33) = −3.94, *p* < 0.001) as measured by a scale commonly used in studies of the effects of nature-based activities.

### 3.4. Holistic Well-Being

We used a biopsychosocial-spiritual framework to assess well-being before and after the camp (Table 6). In the physical domain, participants did not significantly change their activity level (*t*(35) = −1.78, *p* = 0.084), but they did feel significantly more relaxed (*t*(34) = 2.34, *p* = 0.025). This was paralleled by the only significantly changed psychological measure, perceived stress, which was reduced (*t*(35) = 2.45, *p* = 0.020) after camp. In the emotional domain, positive affect increased (*t*(30) = 4.25, *p* < 0.001), while negative affect decreased (*t*(34) = −3.23, *p* = 0.003). The social measure “Positive Relations With Others” approached but did not reach statistical significance (*t*(34) = −1.90, *p* = 0.066). Both measures of spiritual well-being, wholeness and experience of transcendence, significantly increased (*t*(34) = −2.66, *p* = 0.012, and *t*(34) = −3.36, *p* = 0.002, resp.).

Surprisingly, several psychological measures, including resilience (*t*(33) = −1.79, *p* = 0.083), psychological well-being (*t*(34) = −0.071, *p* = 0.943), self-esteem (*t*(34) = −0.06, *p* = 0.950), self-awareness (*t*(35) = −1.30, *p* = 0.200), and reflection (*t*(34) = 1.56, *p* = 0.129), showed no significant change. This may reflect the relatively high precamp scores on these measures, leaving little room for increase due to the camp experience.

### 3.5. Correlations

Two interesting constellations of relationships were identified through examination of the correlations ≥ ±0.50 (all *p* < 0.01; Table 7). Higher change scores on exposure to nature were correlated with improvement in social well-being. Greater changes in social relations and nature-based skills were associated with a willingness to lead in a nature setting. Gaining nature-based skills was also associated with feeling a greater sense of safety which was itself strongly associated with the sense of place measures, that is, developing a greater attachment to natural spaces and a greater sense of continuity of self from nature.

In the second constellation, increased nature connection is associated with decreased perceived stress. The associated changes in perceived stress and relaxation are each related to increased positive emotions and decreased negative emotions, although the emotional poles are not strongly associated with each other. Elevation of self-esteem is also associated with improvement in positive emotions.

### 3.6. Qualitative Results Exploring Social Relationships

While quantitative assessment of social well-being showed no significant change (*M* = 74.17, SD ±12.39 versus *M* = 77.40, SD ±13.28; *p* = 0.066), the ranking questions emphasized the enjoyment and importance of social interactions with peers and the correlations identified a positive relationship between nature exposure and social well-being. Findings from the qualitative interviews emphasized the process of making friends, the importance of being part of a group, and how the wilderness environment facilitated interpersonal connection.

Making friends was enhanced by spending time together, listening to each other, developing intimacy, and breaking social expectations. Quotes around making friends included “everyone has a marvelous story to tell… be patient and listen” and “everyone is like - really different and really accepting of everything each other does - which is really different from home where we have little cliques.” Another stated, “I hugged a lot of people this summer.” One person summed it up with, “You have friends you will keep for a long time.”

Being part of a group included subthemes of a sense of community, engaging in group play, absence of privacy, working together as a team, and the bonding effect of shared experiences. Exemplar quotes included “… [The] community that forms here is remarkable.” The group play was highlighted with, “We lay out on the green and just play cards and then, at night, we just sit on the benches and talk.” Teamwork and the bonding of the team was further explained, “Always a team effort.” “Our crew was really well bonded. We knew each other. We knew all of our strengths and weaknesses… We knew what we should not do around others to make them get ticked off at you.” Another commented on the benefit of “having comrades in your excitement and discomfort.” Ultimately this built a sense of belonging “because you feel like you belong somewhere, you feel like this is where you're supposed to be and self-esteem has gone up.”

The natural environment specifically enhanced the processes of making friends and being part of a group through loss of ego and vanity, breaking down barriers, and limited distractions. This facilitated deeper relationships than would occur in urban surroundings and afforded social relatedness, positive feelings, and a sense of interdependence with others. Some quotes supporting how nature particularly influenced their social experiences included “you're outside. You're cut off so it's a lot easier to get to know people”; “the space forces us to grow closer… the type of space we're doing it in forces us to do it together”; “our connections that we made outside were much deeper than the ones made in an urban setting”; and “I think [being in wilderness] made me more outgoing and less worried about things like what I'm wearing - I do not care anymore - how I look. I think it makes my priorities different.” A final comment reflected on the priority of human interaction over technological interaction: “to look to people first before…Google.”

Being in the natural environment, away from the usual urban setting with its distractions (e.g., continuous internet access), provided a space for human-to-human interaction, allowing friendship to grow. The challenges of being in the wilderness fostered teamwork, enhancing the sense of community. The young people in this camp appear to have made deep and lasting connections and began to feel like they truly belonged.

## 4. Discussion

Findings demonstrate the change in relationship to nature that an immersion experience in wilderness can provide while also delineating elements of well-being that can be affected during time spent in nature.

Measuring multiple aspects of the experience of and relationship with nature can begin to parse what may be needed to address “nature-deficit disorder.” Prior to camp, our study participants' nature connection scores were similar to other early college-aged math, chemistry, or psychology students [44]. The young people in our study found camping activities to be difficult yet ultimately among the most rewarding and influential parts of their experience. The quantitative measures document increased nature exposure, skill level and knowledge, sense of safety, connections with the natural environment, and concomitant willingness to take a leadership role. Further, after camp, their nature connection scores changed to become more similar to environmental studies students [44]. These findings point toward a mitigation in feelings of disconnection, providing insight into what might be needed to bring people into greater comfort and connection with nature. Our results provide further support for suggestions by others that wilderness camping could be an intervention for “nature-deficit disorder” [38]. Given previous research [44, 60], that has demonstrated a link between various measures of connection to nature and environmentally friendly behavior, there also could be an extended role for camps in preparing environmental citizens for our increasingly urbanized and ecologically challenged world.

Through incorporation of multiple measures of well-being, we were able to identify the holistic health benefits associated with a nature immersion experience: more relaxation, less perceived stress, improved emotional states, and more experience of spiritual well-being. The exploratory correlations illustrate a link between nature connection and perceived stress which itself was associated with relaxation and both positive and negative emotions. Our own correlational finding are similar to Howell et al., who studied young college students and found that nature connection correlates with mindful awareness [61], emotional well-being, and happiness index [62]. Capaldi et al.'s recent meta-analysis demonstrates, across over 8000 subjects, a persistent small effect size for the correlation of nature connectedness and measures of happiness, including positive emotions [8]. These interlinked relationships are theoretically supported by Ulrich's psychoevolutionary model [12, 63], which states that interacting with nature initiates a series of physiological, emotional, cognitive, and behavioral responses that result in stress recovery, physiological relaxation, more positive affect, and reduced negative affect. The model correlates with physiologic findings from the study of the Japanese practice of “forest bathing” that document lower cortisol levels, lower sympathetic activation, and greater parasympathetic nervous activity in nature compared to urban environs [34]. Relaxation has been shown to buffer stress and emotional problems [64–66] and can be produced by various health practices (hypnosis, meditation) as well as by the tradition of nature mystics, who immerse themselves in the quiet of nature [67]. Positive emotions are important as they have been related to overall positive health effects, such as human psychosocial flourishing [68] and longevity [69, 70]. 

Nature's effects on spiritual well-being have been previously explored qualitatively [36, 37] but here are documented through use of quantitative scales that extend previous work [39] and may be useful in further studies. While we incorporated numerous measures of psychological development, unlike the wider literature on camp experience [24], this study found no change. This is likely due to high levels of development of the participants.

Surprisingly, no significant change was found on the quantitative assessment of social well-being. Yet the importance of spending time with friends was ranked highly and social relationships were clearly emphasized in the interviews, including the process of making friends, the value of being part of a group, and the wilderness environment's facilitative role in making those connections. This pattern of findings parallels those of researchers investigating group walks in nature whereby quantitative measures show no effect on social well-being [15] while qualitative studies document the importance of social connections [71].

### 4.1. Limitations

The inferences that can be drawn from this study are limited by the small sample size and by the lack of a control group. The 78% response rate for the postquestionnaire is not as robust as might be hoped, although there were no differences between responders and nonresponders. We did not collect complete socioeconomic data, as that was felt to be intrusive to the participants, so analysis of that type of data as a predictor of effect could not be done. Our well-being results could have been confounded by the reduced electromagnetic radiation within the US Radio Quiet Zone; however, the lack of cell phone connectivity outside the camp may have facilitated greater connection with nature and others present in the camp which we would identify as a strength of the study. We also need to be cautious about interpretation given multiple outcome measures. Since this was conceived as a pilot study, exploration of multiple measures was purposeful, allowing us to parse various parts of nature exposure, nature connection, and human well-being. Moreover, an appropriate control group, such as participants in a residential urban educational and recreational program, could strengthen the claim of the association of nature contact with the improvement in wellbeing experienced. We also are aware that physical activity was measured with a single item that did not provide details about the duration and intensity of exercise which might have revealed a change while in the camp setting. Finally, our ability to detect or measure change in the putative “nature-deficit disorder” is hampered by the lack of diagnostic criteria and a suitable measure. Lower scores on the nature-related measures are, at best, only approximations of disconnection with nature.

### 4.2. Future Research

The exploratory correlations found here suggest relationships that could be assessed in future larger-scale studies. One might hypothesize, for example, that acquiring nature-related skills supports a greater sense of safety in nature, which then allows a greater emotional attachment to and a greater sense of personal identity from nature. Another hypothesis could be that, for those with better relationships with others, the addition of nature-based skills enables them to have the capacity to lead in a natural environment. Additionally, the relationship of nature connection to the central construct of perceived stress, which is itself related to relaxation, more positive emotions, and less negative emotions, would be interesting to explore further in this type of setting. Further, development of a scale to measure “nature disconnection” or “nature-deficit disorder” would help refine the assessment of whether this negatively phrased concept is meaningful in the discourse around nature contact and human well-being.

Further work is also required to explore the effect of nature on social well-being. In this study, qualitative findings support the idea that nature experiences enhance social well-being among young adults. The lack of corresponding evidence from quantitative measures—here and in other studies—suggests that the construct is perhaps poorly measured by existing instruments. Our interview findings could inform development of a measure of social well-being for use in nature settings among youth. Such a measure could be used and tested in further research into the effects of nature-based therapies on young people's well-being.

Future work in the above areas could be pursued through surveying NYSC camper and staff alumni or by extending the study to other camp settings including those for youth with health conditions and youth camps in other countries.

## 5. Conclusions

Our work provides an exploration of the youth nature camp experience and its effects on health and well-being. Nature immersion in a camp setting positively affected the participants' relationship with nature; increased relaxation along with decreased perceived stress; increased positive emotions and decreased negative emotions; increased sense of wholeness and experience of transcendence; and enhanced social interaction. This research supports the holistic health value of being in a natural environment. In an increasingly urbanized world with reduced opportunities for interaction with nature, the role of wilderness camps to provide the acquisition of nature-based skills that facilitate a deeper attachment to nature is more important than ever. Connection to nature appears to be associated with reduced stress and greater holistic health and well-being, thus counteracting the risk of untoward effects from “nature-deficit disorder.” Young people with this type of immersive nature experience will be needed to provide leadership in envisioning and shaping a healthy and sustainable world for the future.

## Figures and Tables

**Table 1:** Constructs and measures used to assess relationship with and experience in nature.

Construct	Measures	Number of items	Response range	Cronbach's alpha^a^	Reference
Nature experience	Exposure	6	1–5	na	Irvine, 2004 [39]
	Knowledge	5	1–5		Ryan, 2005 [40]
	Skills	17	1–6		Kaplan, 1974 [41]
	Leadership	1	1–6		

Safety in nature	Single item	1	1–5	na	Fuller et al., 2007 [42]

Sense of Place	Place attachment	4	1–5	.86	Fuller et al., 2007 [42]
	Continuity with past	5		.84–.85	Dallimer et al., 2012 [43]

Nature connection	Connection to nature	14	1–5	.79–.84	Mayer and Frantz, 2004 [44]

^a^Alphas are from the published literature.

**Table 2:** Constructs and measures used to assess elements of biopsychosocial-spiritual well-being.

Construct	Measures	Number of items	Response range^a^	Cronbach's alpha^b^	Reference
Physical health domain					

Physical activity	Single item	1	0–7	na	Milton et al., 2011 [45]
Relaxation	Single item	1	1–5	na	No reference

Psychological health domain					

Stress	Perceived stress	10	0–4 (0–40)	.75	Cohen and Williamson, 1988 [47]
Psychological well-being	Self-acceptance	15 (3 per subscale)	1–6 (15–90)	.52	Ryff and Keyes, 1995 [50]
	Autonomy			.37	
	Environmental mastery			.49	
	Purpose in life			.33	
	Personal growth			.40	
Self-esteem	Rosenberg Self-Esteem Scale	10	0–3 (0–30)	.77–.88	Rosenberg, 1989 [51]
Resilience	Ego-Resiliency Scale	14	1–4	.76	Block and Kremen, 1996 [52]
Self-awareness	Situational Self-Awareness Scale/Public Subscale	3	1–7	.82	Govern and Marsch, 2001 [53]
Mental restoration	Reflection (modified)	3	1–5	na	Irvine, 2004 [39]; Fuller et al., 2007 [42]; Dallimer et al., 2012 [43]

Emotional health domain					

Emotional state	PANAS-X, Positive and Negative Affect Schedule	10 10	1–5	.85–.90 .83–.90	Watson et al., 1988 [54]

Social health domain					

Social	Positive Relations with Others Scale	14	1–6 (14–84)	.88^c^	Ryff et al., 1994 [58]

Spiritual health domain					

Spiritual well-being	Sense of wholeness	7	1–5	.85	Irvine, 2004 [39]
Transcendence	Mysticism Scale (subset of items)	7	1–5 (7–35)	na	Hood, 1975 [59]

^a^Parentheticals indicate range for summed scale scores.

^b^Alphas are from the published literature.

^c^Ryff, Scales of Psychological Well-Being. Undated.

**Table 3:** Participant characteristics (*n* = 36).

Age	Years
Range	18–31
Mode	18
Median	19

Gender	%

Female	67
Male	33

Role	%

Delegates	69
Staff	31

Home environment	%

Rural	14
Small town	39
Suburban	42
Urban	5

**Table 4:** NYSC activity rankings (*n* = 36).

Activity	Most enjoyable	Most influential	Most intellectually stimulating	Hardest
Sig. (2-tailed)^a^	<0.001	<0.001	<0.001	<0.001
Spending time with friends	1	1	4	10
Overnight hiking/camping trips^b^	2	2	5	4
Interactions with staff and presenters	3	3	2	9
Academic components^c^	4	5	1	5
Exercising at camp^bd^	5	8	7	7
Cabin meetings	6	4	3	8
Time on own in nature^b^	7	6	6	6
Rustic accommodations	8	7	8	2
Dining hall food	9	*t* − 9	9	3
Personal computer time	10	*t* − 9	10	1

^a^Ranking analysis was conducted using the Friedman test (*df* = 9); ^b^nature activities provided by the NYSC program setting: ^c^lectures, seminars; ^d^running, walking, frisbee, and volleyball.

**Table 5:** Nature-related measures paired samples* t*-tests, pre- and postcamp responses.

Measure	*N* ^a^	Precamp mean	SD	Postcamp mean	SD	Sig. (2-tailed)^b^
Nature experience						
Exposure	36	3.14	0.56	3.65	0.28	<0.001
Skill	35	2.99	0.85	3.82	0.54	<0.001
Knowledge	36	2.60	0.59	2.79	0.47	0.018
Leadership	34	3.47	1.48	4.68	1.04	<0.001
Feeling of safety in environment	36	3.64	0.87	4.31	0.58	<0.001
Sense of place						
Attachment	36	4.52	0.49	4.73	0.41	0.035
Continuity with the past	36	3.92	0.71	4.22	0.65	0.003
Nature connection	34	3.49	0.67	3.78	0.71	<0.001

^a^Pairwise deletion of missing data.

^b^Paired *t*-test.

**Table 6:** Holistic outcome measures paired samples *t*-tests, pre- and postcamp responses.

Measure	*N* ^a^	Precamp mean	SD	Postcamp mean	SD	Sig. (2-tailed)^b^
Physical activity	36	4.36	2.05	4.97	1.70	0.084
Relaxation	35	3.23	0.88	3.71	0.71	0.025
Perceived stress	34	20.88	5.07	17.82	5.57	0.020
Psychological well-being	35	80.91	5.48	80.97	6.36	0.943
Self-esteem	35	24.91	3.69	24.94	3.55	0.950
Resilience	34	3.20	0.27	3.28	0.33	0.083
Situational self-awareness	36	4.03	1.48	4.37	1.54	0.200
Reflection	35	3.30	0.50	3.11	0.55	0.129
Positive affect	31	3.73	0.62	4.26	0.56	<0.001
Negative affect	35	1.68	0.59	1.34	0.26	0.003
Positive relationship with others	35	74.17	12.39	77.40	13.28	0.066
Wholeness	35	2.90	0.36	3.11	0.35	0.012
Transcendence	35	28.23	4.72	30.51	4.15	0.002

^a^Pairwise deletion of missing data.

^b^Paired *t*-test.

**Table 7:** Pearson correlations between study variables.

	Variables	Correlations between variables																			
		1	2	3	4	5	6	7	8	9	10	11	12	13	14	15	16	17	18	19	20
1	Exposure	1																			
2	Knowledge	.09	1																		
3	Skills	.12	.18	1																	
4	Leadership	.05	.38^*∗*^	.66^**∗∗**^	1																
5	Safety	.24	.30	.51^**∗∗**^	.39^*∗*^	1															
6	Attachment	.27	.31	.33	.32	.85^**∗∗**^	1														
7	Continuity with past	.15	.23	.56^**∗∗**^	.38^*∗*^	.90^**∗∗**^	.55^**∗∗**^	1													
8	Nature connection	.08	.16	.19	.22	.36^*∗*^	.38^*∗*^	.27	1												
9	Physical activity	−.16	.10	.18	.09	.07	.02	.12	.15	1											
10	Relaxation	−.01	−.08	.20	−.06	.09	.03	.13	.34^*∗*^	.04	1										
11	Perceived stress	−.15	−.19	−.32	−.33	−.44^**∗∗**^	−.37^*∗*^	−.43^*∗*^	−.50^**∗∗**^	−.15	−.56^**∗∗**^	1									
12	Psych well-being	−.18	−.12	.29	.41^*∗*^	.22	.11	.29	.08	.17	−.08	.05	1								
13	Self-esteem	−.21	−.04	.19	.27	−.05	−.11	.09	.13	.12	.19	−.42^*∗*^	.05	1							
14	Resilience	−.05	.16	.22	.33	.18	.15	.17	−.03	−.36^*∗*^	.12	−.02	.05	.16	1						
15	Self-awareness	−.25	−.09	−.13	−.02	.13	−.02	.23	.18	.08	.33	−.23	.31	−.15	−.08	1					
16	Reflection	.13	.29	.22	.37^*∗*^	.25	.23	.21	−.07	.015	.06	−.47^**∗∗**^	.12	.25	.04	−.05	1				
17	Positive emotion	−.16	.02	.29	.42^*∗*^	.43^**∗∗**^	.18	.10	.29	.15	.51^**∗∗**^	****−.53^**∗∗**^	.11	.58^**∗∗**^	.17	.08	.35	1			
18	Negative emotion	−.07	.08	.012	.01	.15	.02	−.13	−.12	.03	****−.56^**∗∗**^	.65^**∗∗**^	.12	−.30	.20	−.41^*∗*^	−.27	****−.53^**∗∗**^	1		
19	Positive relations	.56^**∗∗**^	.29	.08	.55^**∗∗**^	−.07	.44^**∗∗**^	.33	.20	−.24	−.07	−.23	.17	−.13	.05	.07	.43^**∗∗**^	.09	−.03	1	
20	Wholeness	−.03	.08	.09	.02	.19	−.01	.32	.33	−.04	.17	−.41^*∗*^	−.03	.41^*∗*^	.06	−.06	.36^*∗*^	.17	−.23	−.04	1
21	Transcendence	−.20	.15	.13	.29	.12	.08	.13	.13	−.001	.33	−.37^*∗*^	.22	.12	−.16	.09	.47^**∗∗**^	.46^**∗∗**^	−.16	.13	.34^*∗*^

^*∗*^ *p* < 0.05; ^*∗∗*^ *p* < 0.0.

## Abbreviations

NDD: Nature-deficit disorder
NYSC: National Youth Science Camp.
